# Anomaly Detection in Retinal OCT Images With Deep Learning-Based Knowledge Distillation

**DOI:** 10.1167/tvst.14.3.26

**Published:** 2025-03-27

**Authors:** Guilherme Aresta, Teresa Araújo, Ursula Schmidt-Erfurth, Hrvoje Bogunović

**Affiliations:** 1 Christian Doppler Lab for Artificial Intelligence in Retina, Institute of Artificial Intelligence, Center for Medical Data Science, Medical University of Vienna, Vienna, Austria; 2 Department of Ophthalmology & Optometry, Medical University of Vienna, Vienna, Austria

**Keywords:** unsupervised deep learning, anomaly detection, optical coherence tomography (OCT)

## Abstract

**Purpose:**

The purpose of this study was to develop a robust and general purpose artificial intelligence (AI) system that allows the identification of retinal optical coherence tomography (OCT) volumes with pathomorphological manifestations not present in normal eyes in screening programs and large retrospective studies.

**Methods:**

An unsupervised anomaly detection deep learning approach for the screening of retinal OCTs with any pathomorphological manifestations via Teacher-Student knowledge distillation is developed. The system is trained with only normal cases without any additional manual labeling. At test time, it scores how anomalous a sample is and produces localized anomaly maps with regions of interest in a B-scan. Fovea-centered OCT scans acquired with Spectralis (Heidelberg Engineering) were considered. A total of 3358 patients were used for development and testing. The detection performance was evaluated in a large data cohort with different pathologies including diabetic macular edema (DME) and the multiple stages of age-related macular degeneration (AMD) and on external public datasets with various disease biomarkers.

**Results:**

The volume-wise anomaly detection receiver operating characteristic (ROC) area under the curve (AUC) was 0.94 ± 0.05 in the test set. Pathological B-scan detection on external datasets varied between 0.81 and 0.87 AUC. Qualitatively, the derived anomaly maps pointed toward diagnostically relevant regions. The behavior of the system across the datasets was similar and consistent.

**Conclusions:**

Anomaly detection constitutes a valid complement to supervised systems aimed at improving the success of vision preservation and eye care, and is an important step toward more efficient and generalizable screening tools.

**Translational Relevance:**

Deep learning approaches can enable an automated and objective screening of a wide range of pathological retinal conditions that deviate from normal appearance.

## Introduction

The number of patients with vision-related diseases globally has been increasing in the past decades as a consequence of changes in lifestyle and an increase in average life expectancy, with early diagnosis and adequate follow-up being pivotal for reducing morbidity.^1^^–^^9^ For that, patients are first imaged via color fundus photography (CFP) or optical coherence tomography (OCT) and the resulting images are then qualitatively assessed by retinal specialists. This screening effort places a strain on healthcare systems by increasing the workload of ophthalmology specialists, and escalating costs associated with traditional diagnostic procedures.

OCT, in particular, has become the gold-standard imaging modality for vision-related diagnoses.^10^^–^^12^ It offers a fast noninvasive, high-resolution cross-sectional imaging of retinal structures, enabling quantitative assessment of relevant biomarkers such as retinal layer thickness and fluid volume. However, the volumetric nature of OCT further increases the burden of manual assessment. Because of this, there has been an increasing interest in integrating artificial intelligence (AI), namely deep learning (DL) methods, in the clinical practice for automated screening and diagnosis.^13^^–^^16^

Although several methods, namely convolutional neural network (CNN)-based approaches, have already been developed for clinically relevant tasks, including OCT volume-wise classification, retinal layer delineation, and specific biomarker detection and classification,^17^^–^^26^ the translation of these systems to practice is still limited. One of the reasons is that the performance of AI systems tends to drop significantly when exposed to data outside their training domain, while remaining overconfident of their prediction. This is particularly true when handling cases with pathologies outside their training cohort. For this case, a solution would be to collect and curate large datasets for all known diseases, but of course the cost associated with creating such datasets is prohibitive. A viable alternative is thus to handle the problem as an unsupervised anomaly detection task, that is, identifying rare or unusual patterns in the data without relying on labeled examples, using instead intrinsic properties like statistical outliers or deviations from learned norms. In the context of retinal OCT screening, this means detecting abnormal retinal structures or pathologies by training models on normal OCT scans, and flagging scans with morphologies that deviate from this normal baseline. Such approaches enable more general-purpose and robust models capable of identifying a wide range of abnormalities, at the cost of not being tailored to classify specific diseases. In addition, AI methods tend to behave like black-boxes, not providing a clear explanation on why a given decision was made. Indeed, robustness (model accuracy, reliability, and reproducibility) and transparency (in particular explainability) are two corner stones of trustworthy AI,^27^^,^^28^ that is, AI that is legally compliant, ethically adherent, and socio-technically robust. Having such trustworthy systems is essential for their adoption in real-world applications.

The goal of this paper is to develop a robust and trustworthy unsupervised anomaly detection method for retinal OCT capable of identifying cases with abnormal retinal structures without relying on labeled pathological examples. The proposed system can automatically detect OCTs with pathomorphological manifestations, while additionally providing a map showing the location of the identified anomaly.

## Methods

In this work, we approach the issues of model robustness and transparency in the context of automatic retinal OCT screening via the unsupervised anomaly detection paradigm, that is, automatically detecting cases with pathomorphological manifestations without the need for labeling or the presence of such cases in the training data. Specifically, the goal of the system is to learn meaningful representations of healthy cases, and then measure deviations from this expected normality. We thus handle the screening task as an out-of-distribution (OOD) detection problem, that is, the system automatically identifies out-of-distribution samples without any prior knowledge of the characteristics of the anomalies.^29^^–^^33^ Specifically, we utilize a knowledge distillation (KD)-based DL approach^34^ that identifies pathological manifestations outside its training cohort of healthy cases by assigning each sample an anomaly score. The approach also has an inherent explainability, producing maps that pinpoint the location of the anomaly. We validate the system by evaluating its performance at detecting anomalous cases at both volume and B-scan levels, as well as by assessing the association of the predicted scores with disease severity.

### Data Collection and Annotation

#### In-House Dataset

Our method was developed and evaluated using baseline, treatment-naïve, OCT scans from different clinical studies available at the Department of Ophthalmology of the Medical University of Vienna. All the scans were acquired with the SPECTRALIS device (Heidelberg Engineering, Germany) and were of sufficient quality for clinical trial inclusion. Each volumetric scan was assigned to an exclusive class (i) normal, (ii) intermediate AMD (iAMD), (iii) neovascular AMD (nAMD), (iv) geographic atrophy (GA), (v) diabetic macular edema (DME), (vi) Stargardt disease, (vii) retinal vein occlusion (RVO), or (viii) central serous chorioretinopathy (CSC) based on the inclusion criteria of each of the studies that compose these data subcohorts. Scans were considered normal whenever there was a lack of pathomorpholigical manifestations associated with a retinal disease. The dataset is composed of 3247 volumes/eyes from 2713 patients, and we only considered the 5 mm^2^ region around the fovea. The axial resolution of the scans is 3.9 µm and the number of B-scans is in the range of 25 to 261. There are 397 normal and 2850 non-normal volumes. This split was performed study-wise to minimize similarities between the development and evaluation datasets.

All patients gave informed consent prior to inclusion in the respective multicenter clinical trials. Both the respective clinical studies as well as this analysis adhered to the tenets of the Declaration of Helsinki and the standards of Good Scientific Practice of the Medical University of Vienna. This study was approved by the Ethics Committee of the Medical University of Vienna, Vienna, Austria (EK 1246/2016).

#### External Public Datasets

We evaluated the abnormal B-scan detection performance and the association of the system response with disease severity on the training cases acquired with the SPECTRALIS device from the public RETOUCH dataset.^35^ We considered all 24 OCT volumes from 12 eyes/patients with nAMD and 12 eye/patients with RVO acquired with the SPECTRALIS device. Each volume had 49 B-scans with 512 × 496 pixels covering a macular area of 6 mm^2^ with an axial resolution of 3.9 µm. The pathological manifestations of these treatment-naive patients are primarily related to the presence of exudation and fluid. Each volume has manual annotations for intraretinal fluid (IRF), subretinal fluid (SRF), and pigment epithelial detachment (PED).

We complementarily evaluate abnormal B-scan detection on the test set of the publicly available Kermany dataset.^36^ The test set contains 1000 OCT central B-scans equally distributed among normal, iAMD (also known as Drusen), nAMD (also known as CNV) and DME classes, that is, 250 B-scans per class. All samples were acquired with the SPECTRALIS device, but specific acquisition details are not disclosed.

In summary, the distribution of volumes for the different classes of the in-house dataset is shown in the Table. From the normal cases, approximately 70% of the volumes (176 eyes from 176 patients) were used for training the DL-system (see the Table). The number of normal and pathological B-scans in the RETOUCH dataset is 295 and 391, respectively.

**Table.** Number of Eyes/Volumes Per Disease in the In-House Dataset Used for Training and Testing the Anomaly Detection System

Class	Normal	iAMD	nAMD	GA	DME	Stargardt Disease	RVO	CSC	Normal	Non-Normal
Train	279	0	0	0	0	0	0	0	279	0
Test	118	516	738	380	598	130	475	13	118	2 850

### Anomaly Detection With Deep Knowledge Distillation

**Figure 1.**
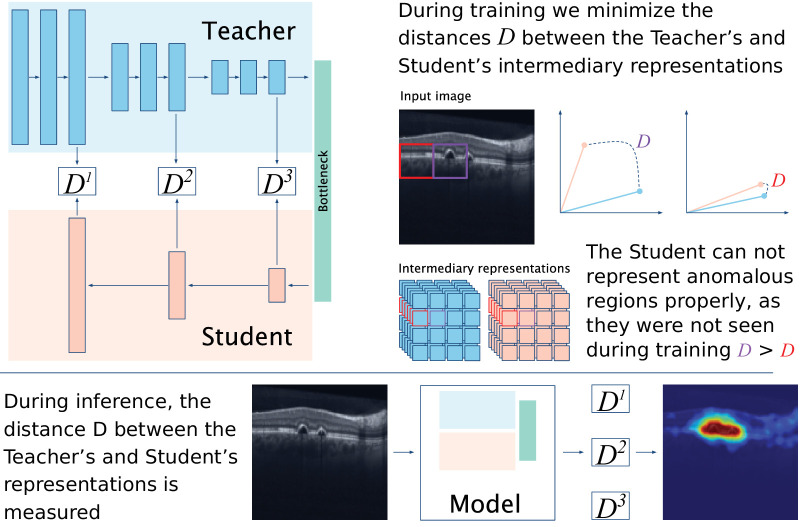
Structure of the studied anomaly detection network. A student model is trained to replicate the intermediate representations of a teacher model when exposed only to normal cases. Differences between the representations allow to measure and localize the anomaly.

The proposed anomaly detection framework (Fig. 1) follows a reverse knowledge distillation paradigm applied to 2D images.^34^ It is composed of three main CNN modules: a teacher (*T*), a student (*S*), and a bottleneck encoder (*B*). *T* is a pre-trained model on a computer vision task, namely classification of natural images from ImageNet,^37^ and thus its parameters allow to extract meaningful intermediary representations (even if not directly related to a specific medical task). *S* is a CNN with a similar architecture of *T*, but with a smaller number of randomly initialized parameters. During training, *T* produces a set of intermediary feature representations, which are combined and simplified by *B*. Then, *S* has to replicate the representations from *T* using as the starting point the summarized information provided by *B*. For anomaly detection in particular, S is trained to replicate T only exposed to normal (non-anomalous) cases.

*T* and *S* have similar model architectures. However, *S* has less intermediary representation capacity than *T* because of the reduced number of parameters. During training, *S* is optimized to replicate the behavior *T* for non-anomalous cases and thus at inference time, the intermediary features of both *T* and *S* should be similar for this type of cases. However, due to the lower capacity and lack of exposure to anomalies on *S*, there will be differences between the outputs of *T* and *S* for anomalous cases. Note that due to the spatial nature of CNNs, each element of the intermediary corresponds to a region of the input image. Thus, measuring the differences in the representations of *T* and *S* at different points of the architecture allows to obtain the relative location of poorly represented regions. This allows us to compute both anomaly maps depicting an approximate location of the anomalies as well as an anomaly score, which summarizes how much a given sample deviates from the learned concept of normality.

#### Definition

Let *X* ∈ R^*H*×*W*^ be the B-scan of size *H* × *W* to assess. Let *T* be a convolutional encoder with *N* ∈ N intermediary levels of interest and corresponding intermediary multi-dimensional feature representations $F_{n}^{T}$, e.g. $F_{2}^{T}=T_{2}\left(T_{1}\left(I\right)\right)$. The weights of *T* are frozen, that is, they cannot be changed during training. The height and width of $F_{n}^{T}$ (*H_n_*,*W_n_*) are a fraction of *H* and *W* dependent on the current intermediary level *n*. *B* aggregates the intermediary outputs of *T* to a single representation, that is, *F^B^* = $B(F_{0}^{T},...,F_{N}^{T})$. Let now *S* be a convolutional decoder with similar architecture to *T*. At each intermediary level *n* ∈ *N*, *S* computes a feature representation $F_{n}^{S}$ with the same dimensions as $F_{n}^{T}$, e.g. $F_{N-2}^{S}=S_{N-2}\left(S_{N-1}\left(B\right)\right)$. During training, we aim at minimizing the distance *D* between all feature vectors pairs of *F^T^* and *F^S^*, that is, $min\sum_{n}^{N}\sum_{i}D\left(F_{n,i}^{T},F_{n,i}^{S}\right)$, where *i* points to each feature vector of the feature maps. During test, we measure the distance between all feature vector pairs of *F^T^* and *F^S^* and retrieve the anomaly scores and anomaly explanation maps as explained below.

#### Distance Metric

In addition,^18^^,^^34^ the distance *D* between the feature vectors is defined as being a weighted sum of the cosine distance and the *L*^2^ distance, as shown in Equation 1:

(1) $$\begin{array}{ccc} & & \;D\left(F_{i}^{T},F_{i}^{S}\right)=\\ & & \;\left[\lambda \left(1-\frac{F_{i}^{T}\cdot F_{i}^{S}}{\max\left(\left|\right|F_{i}^{T}{\left|\right|}_{2}\cdot \left|\right|F_{i}^{S}{\left|\right|}_{2},\varepsilon \right)}\right)+{\left(F_{i}^{T}-F_{i}^{S}\right)}^{2}\right] \end{array}$$

where $F_{i}^{T}$ and $F_{i}^{S}$ are a feature vector from *T* and *S* intermediary representations, respectively, ε is a small number for numerical stability, and λ is a weighting factor. In this work, λ = 1.

#### Loss Function

*S* is trained using a loss based on the distance metric *D*, as shown in Equation 2:

(2) $$\begin{array}{c} L=\sum_{n}^{N}\frac{1}{I_{n}}\sum_{i}^{I_{n}}D\left(F_{n,i}^{T},F_{n,i}^{S}\right) \end{array}$$

where *N* is the number of intermediary feature representations of interest, and *I_n_* = *H_n_* × *W_n_* is the number of feature vectors for the feature map at level *n*.^18^^,^^34^

#### Anomaly Explanation Map

Anomaly explanation maps indicate the approximate location of the regions with higher disparities between the *T* and *S*, that is, the regions where there are likely anomalies. These maps are computed as follows. At each intermediary feature level *n* ∈ *N*, an anomaly score map $M_{n}\in R_{0^{+}}^{H_{n}\times W_{n}}$ is computed via $D(F_{n}^{T},F_{n}^{S})$. All anomaly score maps *M* are resized via bilinear interpolation to the image input size *H* × *W*, summed and smoothed with a Gaussian kernel. The process is repeated independently for each B-scan of an OCT volume, and the resulting maps are concatenated, resulting in a volumetric anomaly map.^34^

#### Anomaly Score

The anomaly score summarizes how much a given input deviates from the learned concept of non-anomaly. We compute the anomaly score by aggregating the differences $D(F_{n,i}^{T},F_{n,i}^{S})$ for each intermediary representation level *n*, that is, $\sum_{n}^{N}P\left(D(F_{n}^{T},F_{n}^{S})\right)$, where *P* is the aggregation function. Unless stated otherwise, we consider *P* = max. Using the maximum as the rule of aggregation instead of global rules, such as the average, allows to more evenly score anomalous cases that result from pathomorphological manifestation of different sizes, for example, drusen and large fluid pockets. The anomaly score is first computed for each B-scan of an OCT volume, and we assign the volume-level anomaly score as the maximum of the B-scan anomaly scores.^34^

All the experiments were performed using Python version 3.9 and PyTorch version 1.12.1, on a workstation with an Intel Core i7-10700K CPU and NVIDIA RTX3080 GPU. Using the workstation, predictions for an OCT volume can be obtained in approximately 0.02 seconds.

### Statistical Analysis

We assume that the studied anomaly detection system has ultimately a binary classification goal (normal versus anomalous), and thus a prediction for a sample (either a B-scan or an OCT volume) can be true-positive (TP) or true-negative (TN) if a case is correctly identified as an anomaly or normal, respectively, or instead false-positive (FP) and false-negative (FN) if the classification is performed incorrectly. The following metrics are used to quantitatively evaluate the performance of the system:

• Positive predictive value (PPV; TP/[TP + FP]) and negative predictive value (NPV; TN/[TN + FN]; both ∈ [0 1]), that is, the proportion predicted anomalous/normal B-scans that are indeed TPs or TNs, respectively. PPV in particular is an important metric in the clinical practice, with higher values indicating a lower risk of a sample being flagged as anomalous while being in fact normal;
• The receiver operating characteristic (ROC) curve, showing the TP rate as a function of the FP rate by varying the decision threshold on the predicted anomaly score;
• The precision recall (PR) curve, that is, the PPV as a function of the true-positive rate (TN/(TN + FP)), also obtained by varying the decision threshold;
• The ROC curve can be summarized by its area under the curve (AUC) ∈[01], with AUC = 0.5 indicating a random predictor, and 1 a perfect classifier. The PR curve can be summarized with the average precision (AP), the weighted mean of the precisions achieved at each threshold, with the increase in recall from the previous threshold used as the weight.

We additionally report correlations using Pearson's and Spearman's rank coefficients. The first evaluates the linear correlation between two measurements, whereas the second assesses the preservation of the ranking of the measurements without considering the relationships to be linear.

### Experiments

#### Detection of Anomalous OCT Volumes

To evaluate the capability of distinguishing between normal and anomalous volumes (TN and TP, respectively), we computed a volume-wise anomaly score (see the Anomaly Detection With Deep Knowledge Distillation section) for each sample of the in-house dataset test set. We then compared the predicted scores from each of the anomalous classes to those from the normal volumes. We also computed the average anomaly profiles across B-scans of a volume to identify the most likely locations of abnormal B-scans for the pathologies in the study. Due to the variability in the number of B-scans per volume, we first linearly interpolated all profiles to the same dimension. Then, we computed the average anomaly score across all volumes at each relative position. Finally, we conducted a qualitative evaluation by assessing the anomaly maps for the different classes of interest.

#### Detection of Anomalous B-Scans

In addition to volume-level detection (see the Detection of Anomalous OCT Volumes section), it is also of interest to understand how well the system can identify anomalous B-scans using the predicted anomaly score. We conducted this experiment using the public RETOUCH dataset and the Kermany dataset. For the RETOUCH dataset, we considered a B-scan to be anomalous if there was at least one manual lesion annotation. Still using the RETOUCH dataset, we further compared the anomaly scores of B-scans without lesions to those with small, medium, and large abnormalities (according to the disease size tercile). Results are evaluated in terms of ROC and PR curves and corresponding AUCs computed for each volume. We additionally computed the PPV and NPV scores for each of the volumes in the RETOUCH dataset via a leave-one-out scheme by repeating the following procedure for each volume: (i) constructing a single ROC curve using the B-scans from all volumes except those from the test volume, (ii) computing the threshold of the optimal cutoff point (the one closest to the point [0,1] in the plot), and (iii) using this threshold to binarize the anomaly scores of the test volume.

#### Association of Anomaly Score With Disease Severity

We further validate our method in the RETOUCH dataset: (i) to assess whether the values of the predicted anomaly maps correlate with the size of the anomalies in each B-scan, and (ii) to confirm that the model behaves appropriately across different disease stages. Our goal is to ensure that the anomaly scores meaningfully reflect the extent of pathological features and that the model is robust in detecting anomalies irrespective of disease progression. For that, we calculated the lesion area, as annotated manually, for each of the B-scans from the RETOUCH dataset. We computed a B-scan-wise anomaly map score by averaging all values of the anomaly map larger than 0.3 (the average anomaly score in the training data), that is, we ignored small activations. We then evaluated the correlation between the lesions’ area (computed as the sum of all IRF, SRF, and PED areas per B-scan) and the anomaly map scores using Pearson's and Spearman's correlation coefficients.

## Results

We evaluated the system in terms of its capability to detect non-healthy OCT volumes (see the Detection of Anomalous OCT Volumes section) and B-scans (see the Detection of Anomalous B-Scans section). We additionally assess the relation between the B-scan-level anomaly scores and the severity of the disease (see the Association of Anomaly Score With Disease Severity section).

### Detection of Anomalous OCT Volumes

**Figure 2.**
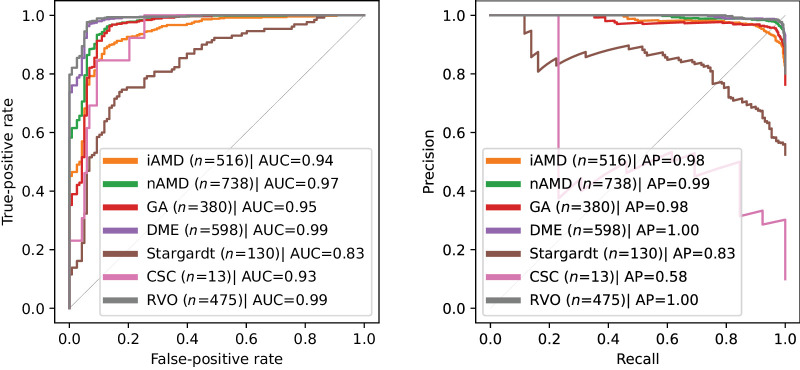
Volume-level anomaly detection performance (normal class versus each of the pathological classes) on the test set of the in-house dataset.

**Figure 3.**
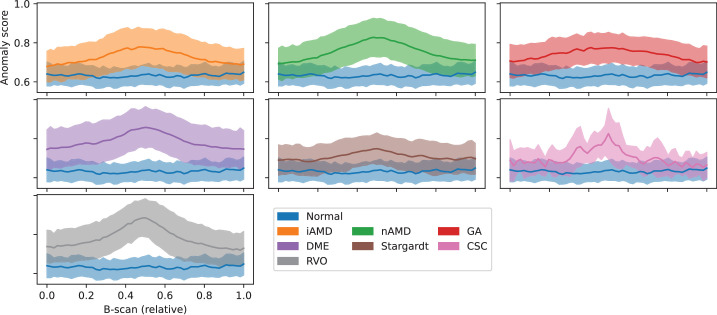
Average B-scan-wise anomaly profiles for the normal class compared with each of the pathological classes on the test set of the in-house dataset.

The ROC and PR curves for the test set of the in-house dataset are shown in Figure 2. The average AUC and AP are 0.94 ± 0.05 and 0.91 ± 0.14, respectively. Results suggest that cases with pathologies that cause higher deformation of the retinal tissue, for example, those with accumulated fluid, are easier to detect. The average B-scan-wise anomaly profiles are shown in Figure 3 and indicate, as expected, a higher lesion presence in the macular center.

**Figure 4.**
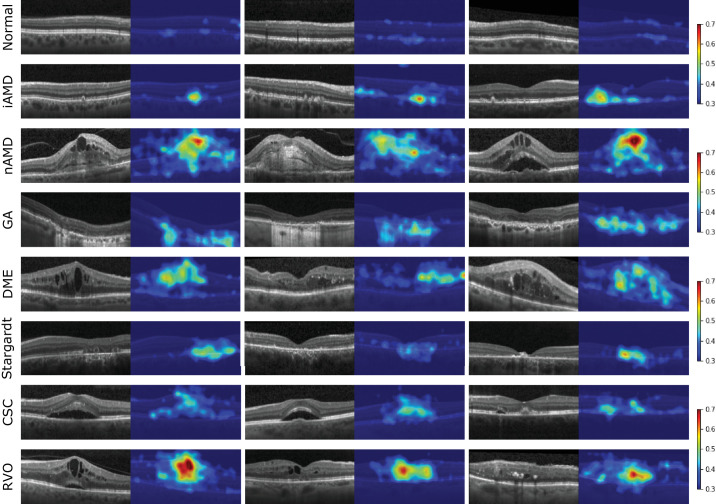
Representative examples of anomaly maps for the different classes in the study (in-house dataset). Colormaps indicate the anomaly score.

Representative examples of the anomaly maps generated by the algorithm for the different classes are shown in Figure 4. Overall, the generated maps are meaningful for retinal diseases of different severity stages. The method is particularly capable of correctly highlighting small to medium pathomorphological manifestations that cause changes in the normal retinal layer structure (see Fig. 4 iAMD and CSC). For large fluid accumulations (see Fig. 4 nAMD and RVO), the system tends not to highlight the fluid itself, but rather the adjacent deformed region.

### Detection of Anomalous B-Scans

**Figure 5.**
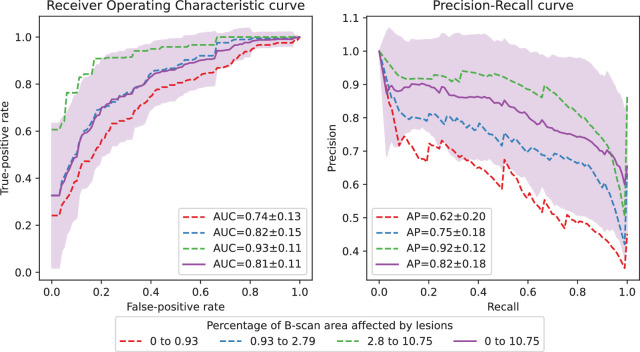
Average volume-wise ROC and PR curves for non-normal B-scan detection in the RETOUCH dataset, considering different levels of pathomorphological manifestations (terciles of the percentage of B-scan area affected by lesions). The *shaded area* corresponds to the standard deviation of the average curves for all volumes.

**Figure 6.**
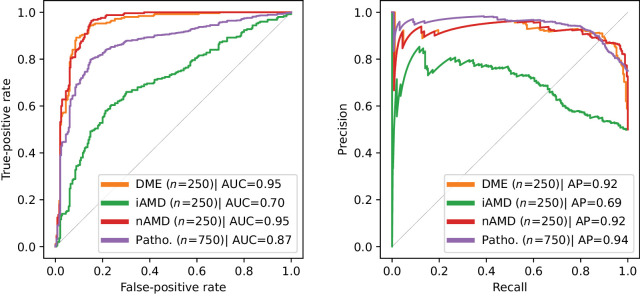
B-scan level anomaly detection performance (normal class versus each of the pathological classes) on the Kermany dataset. Patho. corresponds to all pathological (non-normal) cases considered as a single class.

The capability of the system to detect anomalous B-scans is shown in Figures 5 and 6. The performance for the RETOUCH dataset was computed volume-wise, whereas for the Kermany dataset it was computed for all samples together as only the central B-scans are available. For the RETOUCH dataset, the overall detection ROC AUC was 0.81 ± 0.11 and AP was 0.82 ± 0.18. For the Kermany dataset, the overall pathological B-scan detection AUC was 0.87 and AP was 0.94. The volume-wise B-scan-level PPV and NPV are 0.90 ± 0.11 and 0.55 ± 0.26. The particularly high PPV highlights the robustness of the system in identifying B-scans with diagnostic interest.

### Association of Anomaly Score With Disease Severity

**Figure 7.**
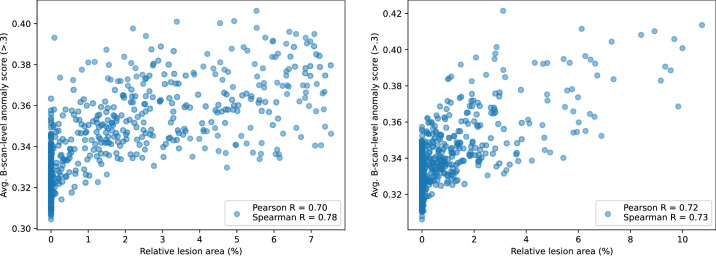
Average B-scan-wise anomaly score as function of the relative area of the manual annotations of IRF, SRF, and PED in the RETOUCH dataset for cases with AMD and RVO.

The results (Fig. 7) show a strong nonlinear ranking (Spearman's rank correlation of 0.78 when considering both AMD and RVO cases) between the scores of the predicted anomaly maps and true lesion size. The correlation was similar for both diseases. The average score of the B scan level anomaly map is therefore a good indicator of disease severity.

## Discussion

This study evaluated a robust DL-based framework designed for automatic anomaly detection in retinal OCT volumes, demonstrating promising results at both the volume and individual B-scan levels. Notably, we conducted an extensive evaluation of the method using a large and clinically relevant OCT dataset, ensuring a thorough assessment of its performance and potential clinical applicability. The method uses an unsupervised knowledge distillation *T –* *S* approach to identify anomalous cases. As a reminder, in this study, we considered an OCT volume as anomalous if it showed signs of retinal disease, which in our test dataset appeared as: iAMD, nAMD, GA, DME, Stargardt disease, RVO, or CSC. The dataset contains cases with different severity stages from each of the diseases.

Automated retinal OCT anomaly detection is an active research topic. For instance, Seebock et al.^38^ proposed to use an uncertainty-aware retinal layer segmentation model trained in normal data to identify potential anomalous OCT regions. In anomalous cases, it becomes difficult for the network to properly segment layers, increasing its uncertainty and thus allowing for anomaly detection. However, this method may miss small anomalies, such as drusen, if the network is still properly capable of segmenting the retinal layers. In addition, weakly supervised approaches are not as future-proof as desirable, because annotation efforts have to be repeated whenever shifts in acquisition settings occur, for example, due to a new acquisition equipment. Because of this, another well-established approach is to use normal data to train generative models.^39^^,^^40^ In essence, these methods work by learning to reconstruct normal B-scans. At test time, when provided with anomalous cases, the networks fail the reconstruction process because they were never exposed to such type of cases. As a consequence, measuring the reconstruction error between the input and generated images allows to infer if a B-scan is anomalous. However, reconstruction-based approaches are notably difficult to train due to their computational demand and need for careful hyper-parameter tuning and potential training instabilities. In addition, although conceptually similar to the approach here presented, which only uses normal data for training, measuring differences in the pixel space, instead of in the feature space, make these approaches very susceptible to noise and reconstruction errors, ultimately increasing the risk of FP detections.

A notable example of an unsupervised retinal OCT detection method is presented by Tiosano et al.^41^ In their work, the authors propose to use a CNN pre-trained in natural images to create a region-level feature set from normal OCT cases. These local region representations are then processed to eliminate redundant samples, creating a representative feature bank. At test time, anomalies are detected by measuring the K nearest neighbors (kNNs) distance between the extracted features and those from the feature bank. Similarly to our approach, having a region-based distance computation allows to produce anomaly explanation maps. However, their method works on a single feature scale, making their out-of-the-box maps coarser. In addition, the performance of these types of multi-step approaches may be highly reliant on hyperparameter selection. Particularly, changes in the number of samples of the feature bank and neighbors during distance calculation can greatly impact the behavior of the approach.

Instead, in this work, we contribute to the existing research on unsupervised retinal OCT anomaly detection by assessing the performance of an easy to set-up end-to-end system that requires no annotation effort (apart from the collection of normal cases) and minimal hyperparameter tuning. The system showed robust detection performance of different pathomorphological manifestations spanning AMD, RVO, and DME. Our results highlight the potential of such types of DL-approaches in enhancing the accuracy and efficiency of retinal OCT image analysis, potentially reducing the burden on clinicians and care providers by providing a reliable preliminary screening tool.

One of the significant advantages of this DL-based anomaly detection system is its unsupervised nature, requiring only data of normal scans for training. Indeed, this approach circumvents the need for a large, labeled dataset of pathological images, which can be challenging and time-consuming to compile. Instead, by training the model exclusively on normal retinal OCT images, it learns to recognize the standard anatomic structures and patterns of the retina. Consequently, any deviation from this learned normality is flagged as an anomaly. Besides the simplification of the model preparation process, especially in terms of data collection and curation, such approach also ensures that the system can generalize to a wide variety of anomalies, including previously unseen pathological conditions. The unsupervised nature of the studied approach allows it to be both adaptable and robust, making it a highly practical solution for real-world clinical settings where the diversity of abnormalities is vast and continually evolving.

By achieving a high detection performance at the volume level, as demonstrated by the 0.94 average volume-level AUC (see the Detection of Anomalous OCT Volumes section, Fig. 2), models such as this one ensure that entire retinal scans can be rapidly and accurately assessed for anomalies, facilitating early detection of various retinal diseases. At the B-scan level (see the Detection of Anomalous B-Scans section), the system's ability to pinpoint specific abnormal slices within a volume allows for more detailed examination and focused clinical attention on the most relevant areas of the dense OCT scan, optimizing the use of clinician time and expertise. In the context of daily clinical practice, this level of automation is essential for managing the increasing volume of retinal OCT scans, driven by a growing and aging population. On the other hand, the B-scan level detection performance was not the same for all types of pathologies. Indeed, we verified that the detection performance is directly related with the amount of pathology present and its size (see the first lesion size in Fig. 5 and iAMD performance in Fig. 6). A possible explanation is that smaller lesions are more subtle and therefore may not be adequately captured by the intermediate features of the teacher model *T*. Nevertheless, the B-scan level detection AUC values >0.8 suggest that this approach can reduce a significant amount of workload in terms of identifying slices of diagnostic relevance.

Following the good B-scan-level anomaly detection performance, one of the standout features of our system is its explainability, which is crucial for clinical adoption. The explanation maps generated by the model provide a visual representation of the detected anomalies, enabling clinicians to understand the reasoning behind the model's decisions (see the Detection of Anomalous OCT Volumes section, in particular, see Fig. 4). This transparency is vital to build trust in the system and facilitates its integration into diagnostic workflows. Moreover, the ability to visualize anomalies directly on retinal OCT images aids in the quick localization of potential issues, streamlining the diagnostic process. We've additionally shown (see the Association of Anomaly Score With Disease Severity section) that the scores of the anomaly maps correlate (Spearman's correlation coefficient >0.7) properly with the amount of disease activity. Because of all of this, producing these explanation maps not only assists in verifying the model's findings but may also enhance the clinician's ability to make informed decisions regarding patient care. On the other hand, the produced anomaly maps still do not properly highlight all pathological manifestations. As shown in Figure 4 nAMD and RVO, large fluid pockets are not fully highlighted. This behavior is most likely due to the similarity of the intensity and noise profiles between large fluid pockets and background. As the method is inherently trained patch-wise, the Student *S* has learned to properly represent low-intensity noisy regions during training (i.e. background) and thus is also partially capable of representing fluid. Despite this, the anomaly maps can still guide the attention of the user to the relevant location within the B-scan, and thus serve as a valuable tool to interpret the algorithm's output.

Unsupervised anomaly detection is a particularly challenging problem, especially in comparison to supervised learning. Unlike supervised methods that rely on labeled samples, unsupervised anomaly detection requires distinguishing unseen pathological patterns from normal variations without any additional information. A key challenge in this task is encoding invariances, that is, determining what the model should be insensitive to. This includes, for example, accounting for variations in acquisition settings, anatomical diversity across individuals, or benign structural changes. This remains an open research problem in medical computer vision. In this work, we focus on a proof-of-concept, demonstrating the feasibility of unsupervised anomaly detection for retinal OCT screening.

The presented work has a few limitations. Being an anomaly detection approach, an obvious pitfall is that the system cannot produce a probability of a specific disease being present in a volume. Indeed, there is a trade-off between the studied *T-S* approach, which is better at performing non-specific screening tasks, and fully supervised approaches, which can be used for accurately diagnosing a known group of diseases while making obvious errors for cases outside their learning curriculum. In the same line of thought, due to its generic anomaly detection capability, the approach may not be capable of distinguishing between anomalies caused by pathologies from those that result from the acquisition pipeline (e.g. noise and artifacts). Albeit not problematic for the studied datasets, in real-world data this limitation may lead to incorrect volume flagging. In addition, the anomaly maps produced by the system are not yet sufficiently comprehensive to enable fully automated detection of pathological biomarkers. In particular, they are not precise enough to pinpoint spatial locations, neither do they allow to differentiate between various types of abnormalities. This limitation makes it difficult to reliably identify the underlying causes of anomalies, especially in cases where the maps highlight regions that are merely distorted or out of place rather than directly indicative of pathological biomarkers, such as fluid accumulation. For example, in large fluid pockets, the receptive field of the model, that is, the effective region of the image the model is assessing, is not large enough to cover the entirety of the pathological region, thus leading to subpar explanation maps. These limitations highlight an exciting opportunity for further work, which can focus on enhancing the system's explainability by incorporating methods that provide more interpretable insights into why certain regions are flagged as anomalous. For example, integrating domain-specific priors, such as the expected thickness of retinal layers, could allow to improve the anomaly maps without sacrificing the generalization capabilities of the system. This would not only improve the system's reliability but also further increase its practical utility in clinical settings, where transparent and interpretable results are essential for effective decision making. Finally, the assessed system considers each B-scan individually and thus does not take advantage of the inherent inter-slice information available in volumetric OCT scans, hindering its ability to produce coherent results for neighboring scans. Incorporating 3-dimensional (3D) information into the system poses several challenges. First, leveraging 3D data significantly increases the computational complexity of the model. In particular, both memory and processing requirements grow because volumetric data involves processing not just individual 2D slices but also their spatial relationships in a 3D context. Such demanding computational requirements are not always available, making the management of 3D medical data a current topic of research in the scientific community. Second, the thickness of slices and their spacing in volumetric scans depend on the acquisition protocol, introducing variability that complicates the extraction of meaningful 3D features. For instance, thicker slices or uneven spacing can result in a loss of fine details and inconsistencies in the volumetric representation, which could degrade model performance. Moreover, the alignment of slices to maintain spatial coherence during pre-processing and model inference is another technical challenge, particularly in cases where motion artifacts or structural distortions are present in the data. To address these issues, future work should focus not only on designing more efficient algorithms capable of processing 3D information but also on extending dataset size and diversity to better capture these complexities and ensure robust model performance across a range of scenarios. In addition, although we evaluate our system in seven different common pathologies, there is a risk that our findings do not generalize to other pathomorphological manifestations. Future research should thus also consider extending the validation of the system to a wider range of diseases. Likewise, the in-house dataset primarily consists of Caucasian individuals, which may limit the generalizability of our findings to populations with different demographic compositions.

Additionally, all data in the in-house dataset were acquired using a single OCT device type (Spectralis), which may introduce certain limitations in applicability when considering other acquisition systems. This is primarily due to potential differences in factors such as noise profiles, field of view, and resolution. However, it is important to note that OCT screening is commonly device-specific, as both the hardware and software configurations of different devices influence the imaging characteristics. Therefore, the use of data from a single device is not an unusually restrictive limitation. Indeed, it is reasonable to expect that the general principles underlying the assessed anomaly detection framework can generalize effectively to other OCT devices with proper fine-tuning or calibration. That is, whereas the in-house dataset reflects the characteristics of the Spectralis-acquired images, the broader approach remains adaptable and applicable to images obtained from other systems.

In conclusion, the presented DL-based anomaly detection system for retinal OCT images shows the potential of unsupervised anomaly detection approaches. The high accuracy at both the volume and the B-scan level ensures comprehensive and reliable anomaly detection, while the anomaly maps provide necessary transparency for clinical use. These features collectively position the system as a valuable tool in the early detection and diagnosis of retinal diseases, potentially improving patient outcomes through timely and accurate interventions. The system's unsupervised nature, requiring only normal data for training, simplifies the data collection process and enhances its ability to detect a wide range of anomalies, including previously unseen conditions. Furthermore, by automating the analysis of large volumes of retinal OCT data, this system has the potential to both alleviate the workload of clinicians as well as increase diagnosis success by providing an objective and explainable second-opinion that could mitigate oversight caused by common factors such as fatigue, time stress, or subjective qualitative assessment. Ultimately, generic approaches such as the one here discussed can constitute a relevant complement to task-specific automated AI-based systems, improving clinical workflow and patient care.
